# Partizipative Forschung mit Menschen mit Behinderungen: Ein Beispiel für die Umsetzung partizipativer Gesundheitsforschung in Werkstätten für behinderte Menschen (WfbM)

**DOI:** 10.1007/s11553-021-00928-8

**Published:** 2021-12-23

**Authors:** Stefanie Gillitzer, C. Thienel, A. Duda, J. Renner, C. Hornberg

**Affiliations:** 1 Fakultät für Gesundheitswissenschaften, Universität Bielefeld, Universitätsstraße 25, 33615 Bielefeld, Deutschland; 2 Medizinische Fakultät OWL, Universität Bielefeld, Bielefeld, Deutschland

**Keywords:** Empowerment, Gesundheitsforschung, Lebenswelt, Gesundheitsförderung, Teilhabe, Empowerment, Health science, Living environment, Health promotion, Inclusion

## Abstract

**Hintergrund:**

Partizipative Gesundheitsforschung (PGF) gewinnt zunehmend an Bedeutung. Durch die Beteiligung der zu beforschenden Zielgruppe können lebensweltnahe, praxisorientierte Ergebnisse generiert und gesundheitsbezogene Veränderungen angestoßen werden. Dies bietet auch Potenziale zur Verbesserung der Teilhabe und Gesundheit von Menschen mit Behinderungen. Viele Forschungsprojekte setzen jedoch nur niedrige Stufen der Partizipation um.

**Ziel des Beitrags:**

Es werden Herausforderungen und entsprechende Lösungsansätze von PGF mit der Zielgruppe Menschen mit Behinderungen diskutiert. Der Beitrag soll Forschenden eine Orientierung zur Erreichung hoher Stufen von Partizipation bieten.

**Material und Methode:**

Es wurde ein Modellvorhaben zur Entwicklung und Umsetzung gesundheitsfördernder Maßnahmen in Werkstätten für behinderte Menschen (WfbM) durchgeführt, das die Erreichung hoher Partizipationsstufen durch PGF mit Menschen mit Behinderungen zum Ziel hatte. Der Prozess wurde durch Interviews und Fragebögen evaluiert.

**Ergebnisse:**

Herausforderungen waren die Diversität der in der Lebenswelt WfbM arbeitenden Menschen, komplexe Strukturen von WfbM, die Verfügbarkeit personeller, zeitlicher und finanzieller Ressourcen sowie lange Kommunikationswege. Der Einbezug der gesamten Lebenswelt, die Nutzung bekannter Kommunikationsmittel und ein häufiger Austausch mit den Beteiligten waren besonders zielführend. Für die Motivation sowie die Verstetigung der entwickelten Maßnahmen war die Wertschätzung gegenüber der Zielgruppe essenziell.

**Schlussfolgerungen:**

Wenn die Herausforderungen von PGF überwunden werden, ermöglicht dies vielfältige Einblicke in die Lebenswelt von Menschen mit Behinderungen, bedarfs- und bedürfnisorientierte Gesundheitsförderung sowie hohe Motivation aller Beteiligten, diese umzusetzen.

## Abstract (en)

**Background:**

Participatory health research (PHR) is becoming increasingly important. Through participation of the target group in research, it is possible to generate real-world and practice-oriented results and to foster health-related changes. This can also serve to improve the participation and health of persons with disabilities. However, the participation levels in many existing PHR projects is low.

**Objectives:**

We discuss challenges and opportunities in PHR with persons with disabilities. This article intends to provide an orientation for achieving high participation levels in health research.

**Methods:**

To develop and implement health-promoting activities in workshops for persons with disabilities, a pilot project was conducted. The aim was to attain high participation levels through PHR with persons with disabilities. The process was evaluated using interviews and questionnaires.

**Results:**

The diversity of the people who are working in the named workshops and especially of the persons with disability was identified as a challenge for PHR. Further challenges are the complex structures and communication lines of those workshops as well as the availability of staff, time and financial resources. Facilitating factors were the inclusion of all stakeholders of this living context, the use of known communication tools and frequent exchange. Appreciation towards the persons with disabilities seems to be one of the most important factors for their motivation and the permanent adoption of health-promoting activities.

**Conclusions:**

If the challenges of PHR are overcome, this enables diverse insights into the lives of persons with disabilities. It facilitates the implementation of health-promoting activities according to the needs of the target group and fosters the motivation of participants.

Partizipation gewinnt in der Gesundheitsforschung zunehmend an Bedeutung. Besonders vulnerable Zielgruppen, wie Menschen mit Behinderungen, können dadurch profitieren. Partizipation ermöglicht eine lebensweltnahe Forschung und Anpassung gesundheitsbezogener Systeme und Maßnahmen. Praxisbeispiele enthalten jedoch oft nur vereinzelt partizipative Elemente und erreichen selten eine hohe Partizipationsstufe. Dieser Beitrag diskutiert Herausforderungen und entsprechende Lösungsansätze partizipativer Gesundheitsforschung (PGF) am Beispiel der Einführung und Umsetzung eines Gesundheitsförderungskonzepts in Werkstätten für behinderte Menschen (WfbM).

Die UN-Behindertenrechtskonvention (UN-BRK) definiert Menschen mit Behinderungen in Art. 1 als Personen, „die langfristige körperliche, seelische, geistige oder Sinnesbeeinträchtigungen haben, welche sie in Wechselwirkung mit verschiedenen Barrieren an der vollen, wirksamen und gleichberechtigten Teilhabe an der Gesellschaft hindern können.“ In Art. 25 der UN-BRK postulieren die Vertragsstaaten, dass diesen ein gleichtberechtigter Zugang zu Gesundheitsleistungen und das erreichbare Höchstmaß an Gesundheit zustehen. Mit dem Behindertengleichstellungsgesetz (BGG) sowie im Gesetz zur Stärkung der Teilhabe und Selbstbestimmung von Menschen mit Behinderungen (BTHG) ist die Gleichstellung dieser Personengruppe auch in Deutschland gesetzlich verankert. Trotz der Bemühungen, ihnen eine umfassende Teilhabe zu ermöglichen, zeigen Studien nach wie vor, dass dies – insbesondere in der Gesundheitsversorgung – nur bedingt gelingt (z. B. [3, 17]). Insgesamt weisen Menschen mit Behinderungen einen schlechteren Gesundheitszustand, ein höheres Risiko für Mehrfach- und Begleiterkrankungen sowie ein schlechteres Gesundheitsverhalten verglichen mit der Allgemeinbevölkerung auf [3, 22]. Die Literatur deutet ferner darauf hin, dass eine mangelhafte Studienlage über die Gesundheit von Menschen mit Behinderungen sowie deren Gesundheitsversorgung besteht. Als problematisch erweist sich hierbei auch, dass es kaum Erfahrungen darüber gibt, welche Formen der Befragung und Forschung geeignet sind. Der Ansatz der partizipativen Gesundheitsforschung (PGF) erscheint hier ideal, um diesbezüglich neue Erkenntnisse zu gewinnen. Einerseits können durch die kontinuierliche Interaktion mit den zu beforschenden Personen schrittweise zielgruppenspezifische Forschungsmethoden entwickelt werden. Andererseits ermöglicht PGF einen tiefen Einblick in die Lebensrealitäten von Einzelpersonen oder Personengruppen, die bei anderen Forschungsmodellen häufig nicht einbezogen werden. Damit birgt PGF die Möglichkeit, gezielt die Gesundheitschancen vulnerabler Gruppen auf Basis von wissenschaftlichen Erkenntnissen zu verbessern [24].

## Partizipation

**Abb. 1:**
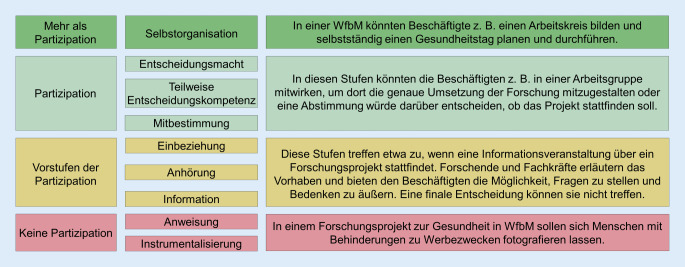
Stufen der Partizipation. (In Anlehnung an Wright et al. [23])

Unter Partizipation wird die Teilhabe von Personen oder Gruppen an Entscheidungsprozessen verstanden [16]. In der Literatur finden sich Synonyme wie Teilhabe, Mitbestimmung, Mitsprache, Einbeziehung, Beteiligung oder Teilnahme. Durch die synonyme Verwendung dieser Begrifflichkeiten lassen sie sich nur schwer voneinander abgrenzen. Obwohl v. a. der Teilhabebegriff im Kontext der Partizipation von Menschen mit Behinderungen genutzt wird, bedarf es hier einer klaren Unterscheidung. Zentrale Aspekte von Partizipation liegen in der aktiven Mitbestimmung, -gestaltung und Einflussnahme auf Entscheidungsprozesse und Ergebnisse, die der Begriff der Teilhabe nicht umfasst [12]. Das Konzept der Partizipation stammt ursprünglich aus dem politischen Kontext und verfolgt das Ziel, Bürger*innen am politischen Geschehen zu beteiligen und ihnen, z. B. durch Volksentscheide und Räte, eine direkte Mitbestimmung zu ermöglichen [7]. Das Verständnis von Partizipation, das diesem Beitrag zugrunde liegt, wird anhand des Stufenmodells in Abb. 1 mithilfe von Praxisbeispielen erklärt [23].

### Partizipative Forschung

Zunehmend gewinnt die partizipative Forschung im Rahmen von gesundheitsbezogenen Fragestellungen an Bedeutung. In der Literatur findet hier der Begriff PGF Verwendung. Oftmals stehen sozial benachteiligte Gruppen, wie Menschen mit Behinderungen, im Fokus der Untersuchungen. PGF eröffnet einen Zugang zu diesen Gruppen und kann so zum Erkenntnisgewinn sowie daraus folgend zu zielgruppenspezifisch angepasster Gesundheitsförderung beitragen [18]. Partizipative Forschung beteiligt explizit die zu beforschende Zielgruppe als Co-Forschende und ermöglicht dadurch, Erfahrungen, Bedürfnisse und Bedarfe der Zielgruppe in einer Untersuchung zu berücksichtigen und bietet eine gute Grundlage zur Entwicklung neuer Lösungsstrategien [1, 5, 13]. Der Begriff Co-Forschende stellt die Zielgruppe mit den Forschenden nahezu gleich und verdeutlicht damit die genannten Ziele der partizipativen (Gesundheits‑)Forschung. Ein Ziel der partizipativen Forschung ist daher, dass allen Beteiligten eine gleichberechtigte Teilnahme, die Möglichkeit zur freien Meinungsäußerung sowie Entscheidungsmacht zugesprochen werden. Entsprechend der Darstellung in Abb. 1 umfasst dies die Vorstufen der Partizipation, tatsächliche Partizipation und eine über Partizipation hinausgehende Teilhabe. Forschung kann dadurch die tatsächliche Lebenswelt der Zielgruppe und praxisnahe Ergebnisse abbilden [1, 5]. Neben dem Erkenntnisgewinn ist ein weiteres Ziel von PGF, bereits durch den Forschungsprozess positive Veränderungen zu bewirken (z. B. Wissensgewinn oder Verhaltensänderung seitens der Zielgruppe; [10]).

### PGF in der Praxis

Während Ansätze der PGF international bereits etabliert sind, finden sich in Deutschland erst seit einigen Jahren zunehmend Forschungsprojekte in diesem Kontext. Eine aktuelle Bestandsaufnahme zur PGF in Deutschland zeigt, dass es eine Vielzahl an Aktivitäten gibt, diese jedoch in der Regel keine Partizipation oberhalb der Stufe *Einbeziehung* aus dem Stufenmodell beinhalten [5]. Auffällig ist dabei, dass methodisch häufig nicht ausreichend beschrieben wird, inwiefern die durchgeführten Studien und Projekte tatsächlich partizipativ stattfinden. Auch in internationalen Projekten ist oft nicht eindeutig, auf welcher Stufe die Partizipation einzuordnen ist. Zum Teil legen Forschende das methodische Vorgehen in Bezug auf partizipative Prozesse detailliert dar und beschreiben in der Ergebnisdarstellung ausführlich, inwiefern die Beteiligten partizipativ eingebunden waren [6]. In diesen Fällen wird deutlich, auf welcher Stufe des Stufenmodells die Partizipation im Projekt eingeordnet werden kann. Häufiger jedoch werden in Veröffentlichungen Teilaspekte der Projekte beschrieben und nur für diese dargelegt, inwiefern Partizipation stattfindet [14]. Damit bleibt für die Lesenden in der Regel unklar, inwiefern die Co-Forschenden darüber hinausgehend partizipativ einbezogen wurden. National wie international ist häufig erkennbar, dass Partizipation v. a. genutzt wird, um die Sichtweisen der Betroffenen einzubeziehen. Dies geschieht z. B. durch Prozesse, in denen Erfahrungen ausgetauscht und reflektiert werden. Darüber hinaus soll Partizipation in vielen Fällen dazu beitragen, den Forschenden ein besseres Verständnis für die Lebenswelt der Co-Forschenden zu ermöglichen [15]. Diese Aspekte sind nach dem Stufenmodell allerdings lediglich als Vorstufen der Partizipation zu bewerten.

Zusammenfassend wird deutlich, dass PGF in vielen Forschungsprojekten zwar adressiert, jedoch nicht konsequent umgesetzt wird. Häufig ist die beschriebene Zielsetzung lediglich als Vorstufe von Partizipation zu bewerten. Tatsächliche PGF (mindestens auf Stufe Mitbestimmung) findet zwar zunehmend, aber insgesamt noch zu selten statt.

Dieser Artikel soll einen Beitrag zum Partizipationsdiskurs leisten und anderen Forschenden eine Orientierung zur Erreichung hoher Stufen der Partizipation in Projekten bieten. Anhand eines Praxisbeispiels aus der Zusammenarbeit mit Menschen mit Behinderungen in Werkstätten für behinderte Menschen (WfbM) werden Herausforderungen und entsprechende Lösungsansätze von PGF diskutiert. Es ist davon auszugehen, dass diese Erfahrungen auch auf andere Lebenswelten von Menschen mit Behinderungen übertragbar sind.

## PGF in WfbM – ein Praxisbeispiel

Da in der Literatur bisher wenige Projekte mit hohen Stufen der Partizipation beschrieben werden, wird im Folgenden ein Projekt vorgestellt, das u. a. zum Ziel hatte, während des gesamten Forschungsprozesses eine hohe Stufe der Partizipation sowie Elemente, die über Partizipation hinausgehen (vgl. Abb. 1*Mehr als Partizipation*), zu erreichen. Diese Erfahrungen sollen dazu beitragen, bestehende Hemmschwellen gegenüber PGF zu senken und Herausforderungen in der Praxis begegnen zu können.

Vor dem Hintergrund des Präventionsgesetzes sowie auf Grundlage des § 20g SGB V wurde ein Modellvorhaben mit dem Ziel der Prävention und Gesundheitsförderung in WfbM durchgeführt. Im Fokus stand die kontinuierliche partizipative Beteiligung der Zielgruppe Menschen mit Behinderungen als Beschäftigte der WfbM an allen Arbeitsphasen (Bedarfserhebung, Maßnahmenentwicklung, Umsetzung und Evaluation) des Vorhabens. Entsprechend der Handlungsfelder – Bewegung, Ernährung, Stress und Sucht – des GKV-Leitfadens Prävention [9] wurden in zwei Modellwerkstätten mit insgesamt elf unterschiedlichen Arbeitsbereichen gesundheitsfördernde Maßnahmen auf Verhaltens- und Verhältnisebene lebensweltorientiert entwickelt und umgesetzt. Durch die Projektvorstellung im Werkstattrat (Beschäftigtenvertretung), bei den Fachkräften sowie durch Aushänge in allen Arbeitsbereichen der Modellwerkstätten und ergänzende E‑Mails an die jeweiligen Arbeitsbereiche waren alle Beschäftigten eingeladen, sich zu beteiligen.

### WfbM als Setting für PGF

Nach § 219 SGB IX sind WfbM Einrichtungen zur Teilhabe am und Eingliederung in das Arbeitsleben für jene Personen, die aufgrund einer Behinderung „nicht, noch nicht oder noch nicht wieder auf dem allgemeinen Arbeitsmarkt beschäftigt werden können“. Ihr Auftrag ist u. a. die Verbesserung oder Erhaltung der Leistungs- und Erwerbsfähigkeit (§ 219 SGV IX). Daraus ergeben sich einige Besonderheiten der Lebenswelt WfbM gegenüber anderen Settings. Erfahrungen aus dem hier beschriebenen Praxisbeispiel zeigen, dass die Zielgruppe der Beschäftigten in den WfbM in vielerlei Hinsicht heterogen ist. Dies resultiert zum einen aus den divergenten Behinderungen, die jeweils verschiedene Ausprägungen annehmen können und unterschiedlicher Betreuungsformen und -intensitäten bedürfen. Zum anderen variiert die Zielsetzung der einzelnen Personen je nach zukünftigem arbeitsmarktbezogenen Ziel. Um allen Bedarfen und Bedürfnissen der Zielgruppe gerecht zu werden, haben WfbM oftmals mehrere Arbeitsbereiche, in denen die Tätigkeiten und auch die Betreuung (z. B. durch Personalschlüssel oder Profession) variieren. Durch diese Konstellation sind häufig hierarchisch geprägte Strukturen zu beobachten. WfbM bieten durch die Kontinuität in der Gruppenzusammensetzung und den täglichen, mehrstündigen Aufenthalt der Beschäftigten einen niedrigschwelligen Zugang zur Zielgruppe. Darüber hinaus ist die Förderung in WfbM aufgrund ihres rehabilitativen Auftrags sowohl gesundheits- als auch arbeitsbezogen.

### Umsetzung von Partizipation im Projektverlauf

Vor dem Projektauftakt in den Modellwerkstätten, stellte sich das Projektteam zunächst allen relevanten Gremien (z. B. Geschäftsführung, Werkstattrat, einzelne Arbeitsbereiche) vor. Sämtliche Angehörige der Lebenswelt WfbM wurden über die Projektziele und anstehenden Arbeitsschritte informiert. Zudem wurde ihre Zustimmung zur Umsetzung des Vorhabens eingeholt. Eine transparente Kommunikation war im gesamten weiteren Projektverlauf essenziell. Aus diesem Grund fanden in regelmäßigen Abständen Informations- und Abstimmungstreffen statt.

Die Partizipation wurde im Modellvorhaben in Arbeitsgruppen (AG) mit je 15 Teilnehmenden aus der Zielgruppe umgesetzt. Aufgrund einer örtlichen Trennung und den unterschiedlichen Behinderungen (Menschen mit körperlichen und geistigen Beeinträchtigungen vs. Menschen mit psychischen Störungen und erworbenen Hirnschädigungen) wurde jeweils eine Arbeitsgruppe je Modellwerkstatt gegründet. Teilnahmevoraussetzung war lediglich das Interesse an gesundheitsbezogenen Themen. Die angeleiteten AG stellten die Basis für die partizipative Entwicklung gesundheitsfördernder Maßnahmen dar und ermöglichten der Zielgruppe die Mitgestaltung dieser nach ihren Bedarfen und Bedürfnissen. In diesem Sinne kann im Rahmen dieses Praxisbeispiels nicht nur von PGF, sondern auch von partizipativer Interventions- oder Maßnahmenentwicklung gesprochen werden.

### Bedarfserhebung und Maßnahmenentwicklung

Die AG fanden in einem 2‑wöchigen Rhythmus statt und begannen mit einer Bedarfserhebung. Als partizipative und performative Methode wurde Photovoice genutzt, da dazu weder ausgeprägte sprachliche Fähigkeiten noch Lese-Rechtschreib-Kompetenzen benötigt werden [2]. Die Teilnehmenden sollten Dinge in ihrer Werkstatt fotografieren, die sie mit dem Thema Gesundheit verbinden. Die Fotos dienten dann als Diskussionsgrundlage in den AG und für das Sammeln möglicher gesundheitsfördernder Maßnahmen, die im Verlauf weitergehend priorisiert wurden. Die Teilnehmenden selbst stellten der Führungsebene sowie anderen Angehörigen der Lebenswelt (z. B. Küchenpersonal) die priorisierten Ideen vor und diskutierten mit ihnen deren Umsetzbarkeit. Für die Zielgruppe hatte dies eine hohe Bedeutung, weil sie so die Möglichkeit bekamen, sich für ihre gesundheitsbezogenen Interessen einzusetzen sowie ihre Bedarfe und Bedürfnisse zu kommunizieren. Mit Abschluss dieser Arbeitsphase entstanden gesundheitsfördernde Maßnahmen in den Bereichen Bewegung, Ernährung und Stress. Das Thema Sucht wurde von der Zielgruppe nicht priorisiert.

### Partizipative Maßnahmenumsetzung

Alle Maßnahmen wurden so konzipiert, dass sie langfristig ohne Unterstützung durch das Projektteam oder weitere externe Gesundheitsprofessionen in den Werkstätten umsetzbar sind. Dies gelang durch die partizipative Einbindung der Zielgruppe in die Maßnahmenumsetzung. Ziel verschiedener Workshopangebote (z. B. zu Bewegung, Ernährung sowie Stress und Entspannung) war es, die Gesundheitskompetenz der Teilnehmenden zu stärken und sie zu ermutigen, gesundheitsfördernde Maßnahmen selbstständig umzusetzen oder anzuleiten (z. B. regelmäßige Bewegungspausen). Als Hilfe zur Umsetzung erhielten sie außerdem entsprechende Materialien (z. B. ein Heft mit Bewegungsübungen und Hinweisen zur korrekten Ausführung).

In einer zusätzlichen Schulungsreihe wurden einzelne, besonders an gesundheitsbezogenen Themen interessierte Beschäftigte zu Multiplikator*innen fortgebildet. In sechs Terminen erhielten die Teilnehmenden tiefergehende Informationen zu den einzelnen Themenbereichen Bewegung, Ernährung, Stress und Entspannung sowie Sucht und diskutierten deren Praxisbezug. Dieses Wissen sollten sie an andere Beschäftigte weitertragen. Auch sollten sie auf gesundheitsbezogene Themen in ihrer Werkstatt aufmerksam machen und mögliche Bedarfe mit Verantwortlichen besprechen. Im Sinne der Partizipation sollte dadurch tatsächliche Mitsprache und Entscheidungsmacht in den Werkstätten ermöglicht werden.

### Evaluation der gesundheitsfördernden Maßnahmen

Zur Evaluation der implementierten gesundheitsfördernden Maßnahmen sowie des partizipativen Prozess fanden eine quantitative Prä-Evaluation (*n* = 133) und Post-Evaluation (*n* = 65) sowie eine qualitative Prozessevaluation (*n* = 21) statt. Das übergeordnete Ziel war, die Umsetzbarkeit, Nutzung und Akzeptanz der Maßnahmen in WfbM zu evaluieren. Alle Fragebögen bzw. Interviewleitfäden wurden zusätzlich in Leichter Sprache zur Verfügung gestellt und in einem Pretest erprobt. Die Prä-Evaluation diente der Erhebung des aktuellen Stands des Gesundheitsverhaltens und bestehender Maßnahmen. Die Konzeption des Fragebogens war an standardisierten Fragebögen zu den Themen Bewegung, Ernährung, Stress und Entspannung sowie Sucht orientiert, allerdings wesentlich vereinfacht und reduziert, um die selbstständige Beantwortung für die Beschäftigten zu ermöglichen. Zur Überprüfung, ob die implementierten Maßnahmen zu Veränderungen in den WfbM führten, wurde nach einem Jahr der Umsetzung die Post-Evaluation durchgeführt. Ergänzend zum ursprünglichen Fragebogen wurden hier Items zur Häufigkeit der Teilnahme an und der Bewertung von implementierten Maßnahmen gestellt. Die Auswertung erfolgte zunächst deskriptiv, um Vergleiche ziehen zu können. Anschließend diente eine bivariate Analyse (χ^2^-Test) der Identifikation möglicher Zusammenhänge.

Die Prozessevaluation erfolgte bereits in der Phase der Maßnahmentestung. Dies ermöglichte zeitnahe Anpassungen der Rahmenbedingungen und Maßnahmen. Ziel war es, die Maßnahmenumsetzung so zu optimieren, dass höhere Stufen der Partizipation erreichbar wurden. Fördernde und hemmende Bedingungen für die Implementierung der gesundheitsfördernden Maßnahmen wurden ermittelt und daraus Handlungsempfehlungen für andere Werkstätten abgeleitet. Darüber hinaus stand die Identifikation von Anpassungsbedarfen und aktuellen Herausforderungen in der Maßnahmenumsetzung im Fokus. Teil der Prozessevaluation war auch die Evaluation der Partizipation innerhalb des Projekts, die durch einzelne Fragen im Leitfaden adressiert wurde. Es nahmen 17 Beschäftigte (B) und 4 Fachkräfte (FK) an den teilstrukturierten Interviews teil.

## Herausforderungen und Lösungsansätze für PGF mit Menschen mit Behinderungen

Anhand der quantitativen und qualitativen Evaluationsergebnisse ließen sich folgende Herausforderungen und entsprechende Lösungsansätze sowie daraus abgeleitet Erfolgsfaktoren für den Prozess der PGF mit Menschen mit Behinderungen identifizieren.

### Partizipation der gesamten Lebenswelt

In der Lebenswelt WfbM halten sich täglich sehr unterschiedliche Personengruppen auf. Neben den Beschäftigten arbeiten dort beispielsweise pädagogische oder handwerkliche Fachkräfte, Küchenpersonal, Abteilungs- und Geschäftsleitungen. Für die Umsetzung des partizipativen Forschungsprozess barg dies einige Herausforderungen, weil einerseits die Interessen und Bedarfe der verschiedenen Personen/-gruppen stark variierten und andererseits unterschiedliche Kommunikationsweisen erforderlich waren. Für eine erfolgreiche Umsetzung der Gesundheitsforschung und langfristige Verstetigung der daraus entstandenen gesundheitsfördernden Maßnahmen war die Berücksichtigung all dieser Variablen essenziell. Die Evaluationen sowie informellen Gespräche zeigten, dass das Interesse an der Teilnahme an Veranstaltungen im Rahmen des Modellvorhabens hoch war. Dies sagten sowohl die Beschäftigten als auch die Fachkräfte. Obwohl die gesundheitsfördernden Maßnahmen insbesondere auf die Zielgruppe der Menschen mit Behinderungen ausgerichtet wurden, war es wichtig, diese an der gesamten Lebenswelt zu orientieren.

„Wenn es wirklich für alle und jeden sein soll, dann sollten auch alle und jeder die Möglichkeit haben, daran teilzunehmen.“ (FK2).

Die Partizipation aller Angehöriger der Lebenswelt, wie z. B. der Fachkräfte und verschiedener Gremien, erzeugte eine hohe Akzeptanz gegenüber den neuen Maßnahmen und steigerte zudem die Motivation, sie in den Werkstattalltag zu integrieren. Laut den Interviews war die Unterstützung der Fachkräfte besonders hoch, wenn sie, neben den Beschäftigten, auch an Veranstaltungen des Modellvorhabens teilnahmen. Es wurde zudem deutlich, dass die Fachkräfte im Prozess eine wichtige Rolle bei der Weitergabe von Informationen spielten.

### Kommunikation und Informationsvermittlung

Die Strukturen von WfbM sind oftmals hierarchisch geprägt und komplex. Dementsprechend zeigten sich während des partizipativen Forschungsprozesses Herausforderungen hinsichtlich der Kommunikation und Informationsvermittlung. Lange interne Kommunikationswege und langanhaltende Diskussionen wurden dahingehend als Hindernisse identifiziert.

In den Interviews zur Prozessevaluation gab es ferner den Hinweis, dass zu manchen Zeitpunkten im partizipativen Forschungsprozess nicht klar war, welche Arbeitsgruppen, Workshops etc. für welche Zielgruppe in der Werkstatt angedacht waren. Daraus folgt, dass eine klare Kommunikation und Ansprache der Zielgruppe von großer Bedeutung ist. Um die Zielgruppe der Menschen mit Behinderungen direkt zu erreichen und lange Kommunikationswege über Geschäftsführung, Abteilungsleitung oder Fachkräfte zu vermeiden, wurden die Art, Inhalte und Erreichbarkeit der Kommunikation an die Zielgruppe angepasst. In informellen Gesprächen zu Beginn des Prozesses zeigte sich auch hinsichtlich der Kommunikation die Heterogenität der Zielgruppe. Während einige Beschäftigte häufig via Smartphone oder Computer kommunizierten, waren andere ausschließlich analog erreichbar. Im Rahmen des Projekts wurden demnach überwiegend Flyer oder Aushänge als Informationsmittel verwendet. Daneben dienten auch persönliche Ankündigungen und Gespräche der Verbreitung von organisatorischen Informationen.

„Ja, die Termine waren immer, haben stattgefunden, wann sie angekündigt waren. Man war immer vorher informiert, wenn Termine waren, auch längerfristig, sodass man sich da Termine freihalten konnte und es stand immer dabei, worum es geht, was also in etwa passieren wird, fand also das Informationssystem war ganz gut.“ (B1)

Die frühzeitige Bekanntgabe von Terminen ermöglichte es, diese besser in den (Arbeits‑)Alltag integrieren zu können. Das Augenmerk sollte nicht nur auf der Art der Kommunikationsmittel (z. B. Flyer, Plakate, E‑Mail-Newsletter) liegen, sondern auch auf den Inhalten. Neben einer verständlichen oder Leichten Sprache, sind Angaben über Orte, Zeiten und Ansprechpersonen notwendig. Eine niedrigschwellige Informationsvermittlung mit einfachen Erklärungen, Leichter Sprache und bildlichen Darstellungen zeigte sich auch für gemeinsame Arbeitsgruppen und Workshops als Erfolg versprechend. Die Beschäftigten fühlten sich durch diese Ansprache und den Aufbau der unterschiedlichen Prozessschritte gut in den Forschungsprozess einbezogen. Dies entspricht im Modell zwar Vorstufen der Partizipation, ermöglichte es jedoch im weiteren Projektverlauf die tatsächliche Partizipation der Zielgruppe zu erreichen.

### Forschung auf Augenhöhe und Wertschätzung

Seitens der Forschenden bestand die Herausforderung, von zuvor umgesetzten Forschungsprojekten abzuweichen und die Teilnehmenden nicht als zu beforschende Gruppe, sondern als Co-Forschende zu betrachten. Nur auf diese Weise konnten hohe Stufen der Partizipation erreicht und nah an der Zielgruppe ausgerichtete gesundheitsfördernde Maßnahmen entwickelt werden.

In den Interviews betonten die Beschäftigten, dass das partizipative Vorgehen im Modellvorhaben sich von anderen Projekten, an denen sie bereits mitwirkten, unterschied und bewerteten dies als sehr positiv.

„Dass ihr eigentlich eh uns Beschäftigten eigentlich auch sehr gut dann immer zugehört habt und auch drauf eingegangen seid.“ (B6)

Es war wichtig, die individuellen Bedürfnisse der Zielgruppe sowohl für die organisatorische Planung von Projektschritten als auch für die Gestaltung der Inhalte zu berücksichtigen. Der Forschungsprozess konnte dahingehend kontinuierlich angepasst werden, sodass höhere Partizipationsstufen erreicht werden konnten. Die Interviewergebnisse zeigen, dass den Beschäftigten v. a. wichtig war, dass sie sich ernstgenommen und wertgeschätzt fühlten.

„Dass das auch ein gutes Miteinander in der Gruppe war. Wo ja alle unterschiedlich sind, ne, verschiedene Abteilungen, aber das war ein gutes Miteinander und das war auch irgendwie ja, gut, dass man auch mal so andere Meinungen mal so ungeschminkt mal so bekommen hat.“ (B14)

Die Wertschätzung hatte nicht nur seitens der Forschenden und Fachkräfte, sondern ebenfalls innerhalb der Gruppe einen hohen Stellenwert. Um die Motivation zur Teilnahme zu stärken und den Beschäftigten eine Wertschätzung entgegenzubringen, wurde im Rahmen des Modellvorhabens ein Zertifikat für die erfolgreiche Teilnahme an der Workshopreihe ausgestellt.

### Verstetigung der partizipativen Prozesse und gesundheitsfördernden Maßnahmen

Das gemeinsame Forschen fördert nicht nur die Motivation der Teilnehmenden, ihre selbst entwickelten gesundheitsfördernden Maßnahmen langfristig umzusetzen, sondern vermittelt ihnen dafür auch das notwendige Wissen und Selbstvertrauen. Die Erfahrungen aus dem Modellvorhaben zeigten, dass die Verstetigung partizipativer Prozesse auch durch weitere Faktoren (z. B. Ressourcen der Lebenswelt) beeinflusst wird. Bereits die Umsetzung der PGF stieß häufig aufgrund mangelnder zeitlicher, finanzieller oder personeller Ressourcen innerhalb der Werkstatt auf Herausforderungen. Einige Arbeitsbereiche der Werkstätten stimmten einer Teilnahme am Modellvorhaben grundsätzlich nicht zu. Gründe waren vorwiegend mangelnde zeitliche Ressourcen der Fachkräfte oder die Teilnahme an parallelen Angeboten. Auch hinsichtlich der Verstetigung der Prozesse wurden diese Bedenken in den Interviews geäußert.

„Leider wahrscheinlich finanziell nicht möglich. Vielleicht kann [die Werkstatt] sich auch vorstellen, dass die da jemanden für abstellen oder zwei. Einer alleine wird es wahrscheinlich nicht schaffen. Vielleicht zwei, die dann das ein bisschen am Laufen halten.“ (FK3)

Es zeigt sich, dass zwar der Wunsch nach einer Verstetigung der Prozesse seitens der Fachkräfte vorhanden war, diese sich aber speziell für diese Aufgabe Unterstützung wünschten. Eine Finanzierung weiteren Personals zu diesem Zweck wurde aber für eher unwahrscheinlich gehalten. Um dieser Problematik entgegenzuwirken, war es essenziell, die Zielgruppen in den gesamten Prozess partizipativ einzubeziehen. Je intensiver die Beschäftigten in den gesamten Arbeitsprozess einbezogen waren, desto höher war die Wahrscheinlichkeit, dass sie die entwickelten Maßnahmen selbstständig und ohne Unterstützung von Fachkräften durchführten und erweiterten, was der höchsten Partizipationsstufe im Stufenmodell, der Selbstorganisation, entspricht. In dieser Stufe erfolgt die Übergabe tatsächlicher Entscheidungsmacht an die Menschen im Setting. Dies wurde auch in der quantitativen Evaluation deutlich, insbesondere an der neu eingeführten Bewegungspause, die häufiger stattfand und besser besucht war, wenn sie von Beschäftigten selbst angeleitet wurde. Informelle Gespräche am Ende des Projekts zeigten, dass die gesundheitsfördernden Maßnahmen auch eineinhalb Jahre nach Einführung noch umgesetzt wurden. Auch während der COVID-19-Pandemie, die den Werkstattablauf maßgeblich beeinflusste, führten die Beschäftigten die Maßnahmen weiterhin durch.

## Diskussion

Ziel des vorliegenden Artikels war es, die Herausforderungen und entsprechende Lösungsansätze der PGF für die Einführung und Umsetzung von Gesundheitsförderung in WfbM anhand eines durchgeführten Modellvorhabens aufzuzeigen.

Herausforderungen für PGF resultierten aus Besonderheiten in Abläufen und Strukturen der Lebenswelt WfbM sowie aus der Diversität der Zielgruppe (z. B. durch unterschiedliche Beeinträchtigungen; [13]). Daher war ein zielgruppenspezifisches Konzept notwendig, das durch den Einbezug der Betroffenenperspektive an Passgenauigkeit und Akzeptanz der zu entwickelnden gesundheitsfördernden Maßnahmen gewinnen konnte. Für viele Menschen mit Behinderungen ist es beispielsweise ungewohnt, selbstständig Entscheidungen zu treffen, da sie oftmals durch Angehörige oder Betreuungspersonal fremdbestimmt sind [11, 13]. Diese Herausforderung bietet jedoch gleichzeitig auch eine Chance: Einerseits müssen seitens der Forschenden zwar Strategien entwickelt werden, um die Zielgruppe möglichst gleichberechtigt involvieren zu können, andererseits können durch partizipative Forschungsprozesse die Selbstwirksamkeit und Entscheidungskompetenz von Menschen mit Behinderungen gestärkt werden. Diese Fähigkeiten können in Kombination mit neuem Wissen über gesundheitsbezogene Themen zudem die Gesundheitskompetenz verbessern [2, 21].

Die PGF unterscheidet sich von anderen Forschungsvorhaben v. a. deshalb, weil die Erreichung von tatsächlicher Partizipation wesentlich intensivere Kommunikation zwischen den Forschenden und der Zielgruppe benötigt. Um eine hohe Stufe der Partizipation erreichen zu können, sollte die Zielgruppe nach Möglichkeit in alle Schritte, inklusive der Auswertung und Interpretation der Ergebnisse, involviert sein. Es ist daher wichtig, dass die Forschenden die Zielgruppe gut kennen, um Inhalte sowie den Prozess an ihr auszurichten. Informationen müssen möglichst transparent sowie für die Zielgruppe zugänglich und verständlich aufbereitet sein, damit die Beteiligten sich gut einbringen können [1, 4]. Die Verwendung Leichter Sprache und einfacher Erklärungen trug im Modellprojekt wesentlich dazu bei. Ebenfalls zielführend waren die Präsenz im Setting (z. B. regelmäßige Termine) und der stetige Kontakt zur Lebenswelt. Um trotz der hierarchischen Strukturen der WfbM alle Ebenen zu beteiligen und partizipativ einzubinden, wurden Informationen auf unterschiedlichsten Wegen vermittelt. In Bezug auf diese Aspekte wurde im gesamten Projekt mindestens die Partizipationsstufe *Einbeziehung* erreicht. Darauf aufbauend konnten dann im Prozess regelhaft die Stufen *Mitbestimmung, Teilweise Entscheidungskompetenz* bzw. *Entscheidungsmacht* erreicht werden. Alle drei Stufen entsprechen tatsächlicher Partizipation im Sinne des Modells. In einzelnen Bereichen konnte darüber hinaus auch die Stufe *Selbstorganisation* erreicht werden, die über Partizipation hinausgeht.

Von Unger (2012) beschreibt eine gleichberechtigte Beteiligung der Zielgruppe als Idealfall partizipativer Forschung [20]. In der Literatur wird ebenso davon gesprochen, dass Machtverhältnisse reflektiert und letztlich reduziert werden müssen, um eine hohe Partizipationsstufe (z. B. Entscheidungsmacht) erreichen zu können [13]. Im dargelegten Modellvorhaben wurde diesbezüglich die Wertschätzung der Menschen mit Behinderungen als Co-Forschende als ein entscheidender Erfolgsfaktor identifiziert. Durch den fortwährenden Informationsfluss, den niedrigschwelligen Zugang sowie die Würdigung der Teilnahme am Forschungsprozess durch Zertifikate fühlten sich die Beschäftigten dem Projektteam gleichwertig.

Die Beschäftigten und Fachkräfte sollten gleichermaßen darin geschult werden, partizipativ zu handeln, um nachhaltige Prozesse zu etablieren [8]. Die Literatur gibt Hinweise darauf, dass Gesetze sowie institutionelle und gesellschaftliche Strukturen partizipative Forschung bedingen bzw. die Partizipation begrenzen können [8, 19, 20]. Durch die zeitlichen, personellen und finanziellen Ressourcen seitens des Projektteams konnten im Modellvorhaben einige Hindernisse durch begrenzte Ressourcen auf Seiten der WfbM zeitweilig überwunden werden. Es ist jedoch zu erwarten, dass diese institutionellen Grenzen langfristig weiterbestehen. Die partizipative Arbeit mit allen Angehörigen der Lebenswelt kann zur Durchbrechung bestehender Hierarchien beitragen und so Partizipation und Kommunikation innerhalb der Lebenswelt WfbM fördern [8]. Dies ist insofern wichtig, als dass zumindest die Partizipation der Beschäftigten in den Arbeitsalltag integriert werden konnte und zur Verstetigung partizipativer Prozesse sowie der Umsetzung gesundheitsfördernder Maßnahmen dienlich ist. Dies zeigte sich u. a. daran, dass, wenn die Zielgruppe selbst mit der Durchführung von Maßnahmen betraut war, sich die Wahrscheinlichkeit für eine Verstetigung erhöhte. In diesem Zusammenhang sind die Unterstützung und das Engagement von Fachkräften in WfbM nicht zu unterschätzen. Sie sollten solche, über Partizipation hinausgehende Tätigkeiten kennen und zulassen. Letztlich kann Partizipation so auch die Fachkräfte entlasten und somit die Notwendigkeit bestimmter Ressourcen reduzieren. Die durch PGF entwickelten gesundheitsfördernden Maßnahmen zeigen, dass Partizipation die Zielgruppe dazu bestärkt und motiviert, sich für ihre Belange einzusetzen, ihr Wissen steigert und eine langfristige Auseinandersetzung mit gesundheitsbezogenen Themen fördert.

Im Modellvorhaben wurde deutlich, dass die Umsetzung von PGF mit der spezifischen Zielgruppe Menschen mit Behinderungen in der Lebenswelt WfbM erfolgreich umsetzbar ist, sofern sich die Forschenden auf die Gegebenheiten und Angehörigen der Lebenswelt einlassen und den Prozess stets an diesen orientieren. Auch innerhalb eines rückblickend gelungenen Forschungsprozess ergeben sich Herausforderungen, denen begegnet werden muss, um hohe Stufen von Partizipation zu erreichen.

Da es sich bei Menschen mit Behinderungen um eine vulnerable Zielgruppe handelt, gilt es auch bei partizipativen Forschungsvorhaben ethische Fragen zu berücksichtigen. Um den Bedürfnissen der Zielgruppe gerecht zu werden, war im gesamten Projekt eine hohe Flexibilität in der Durchführung der Projektschritte erforderlich. Zum einen wurden zum Erhalt der Konzentrationsfähigkeit viele Pausen während gemeinsamer Termine ermöglicht, zum anderen waren Treffen entweder zeitlich kurz angesetzt oder so konzipiert, dass eine Verkürzung möglich war. Nicht nur im Sinne der Partizipation, sondern ebenfalls vor dem Hintergrund ethischer Überlegungen war es wichtig, der Zielgruppe Teilhabe und damit Selbstbestimmung und Entscheidungsfähigkeit zuzutrauen. Herausfordernd war dies einerseits, weil die Fähigkeiten der Beschäftigten in WfbM häufig unterschätzt werden. Andererseits kann es auch für die Beschäftigten selbst ungewohnt sein, eigenständige Entscheidungen zu treffen. Dies erforderte ein gemeinsames Herantasten an Empowerment und Gesundheitskompetenz. Diesbezüglich bestand die Schwierigkeit, dass aufgrund der hierarchischen Strukturen in der WfbM nicht alle Entscheidungen durch die Beschäftigten selbst getroffen werden konnten und daher Kompromisse sowie intensive Abstimmungen mit den Fachkräften notwendig waren. Im gesamten Praxisbeispiel war das übergeordnete Ziel, allen Beschäftigten Partizipation zu ermöglichen. Die Inhalte wurden daher auch in Leichter Sprache vermittelt. Fraglich ist dennoch, ob nicht Hindernisse für Menschen mit eingeschränkten sprachlichen sowie Lese- und Schreibfähigkeiten bestanden. Um die Rücklaufquote und zugleich die Partizipation zu erhöhen, wurde die Prä-Evaluation durch die Projektmitarbeitenden unterstützt. Ethisch fragwürdig ist an dieser Stelle, dass damit ggf. der Druck zur Teilnahme steigt und die Anonymität der Antworten nicht gewährleistet ist. Aufgrund dieser und weiterer ethischer Bedenken wurde vor der Durchführung des Projektes ein Votum der zuständigen Ethikkommission eingeholt.

Abschließend bleibt festzuhalten, dass PGF insgesamt deutlich mehr Ressourcen als Forschungsprojekte ohne explizit partizipative Anteile benötigt. Dies ist im Praxisbeispiel u. a. durch den hohen Betreuungsaufwand für die Zielgruppe begründet. Darüber hinaus sind hohe zeitliche und personelle Ressourcen zur Konzeption von zielgruppenspezifischen Inhalten (insbesondere bei heterogenen Zielgruppen) sowie für kontinuierliche Absprachen von Terminen notwendig. Diese müssen in der Regel lange vorher geplant und wiederholt sowie über verschiedene Kommunikationskanäle angekündigt werden, um möglichst vielen Menschen Partizipation zu ermöglichen. Entscheidend ist zudem die Offenheit gegenüber den Wünschen der Zielgruppe und damit einhergehend die Bereitschaft zu hoher Flexibilität aller Beteiligten. Tatsächliche Partizipation reduziert die Planbarkeit von Projektschritten und -inhalten bereits im Vorfeld.

Wenngleich PGF einige ethische und praktische Herausforderungen mit sich bringt, bildet sie eine gute Grundlage für zielgruppenorientierte Forschung und weitergehend für die Implementierung passgenauer gesundheitsfördernder Maßnahmen. Das Praxisbeispiel zeigt, dass Herausforderungen mit entsprechenden Maßnahmen vorgebeugt oder entgegengewirkt werden kann, sodass eine erfolgreiche Umsetzung partizipativer Forschung möglich ist.

## Fazit für die Praxis

- Die Umsetzung von partizipativer Gesundheitsforschung (PGF) mit Menschen mit Behinderungen in Werkstätten für behinderte Menschen (WfbM) bietet für die Forschung vielfältige Einblicke in die Lebenswelt einer vulnerablen Gruppe. Die Diversität der Zielgruppe bedarf dazu spezifischer PGF.
- PGF ermöglicht die bedarfs- und bedürfnisorientierte Entwicklung von Gesundheitsförderungsmaßnahmen und trägt zu einer hohen Akzeptanz dieser Maßnahmen in der Lebenswelt bei.
- Kontinuierliche Kommunikation ermöglicht es, trotz komplexer Strukturen in WfbM alle Angehörigen der Lebenswelt zu erreichen und einzubinden. Dies gilt gleichermaßen für die organisatorische als auch inhaltliche Zusammenarbeit mit der Zielgruppe.
- Die Investition zeitlicher, personeller sowie finanzieller Ressourcen zahlt sich langfristig für die Lebenswelt aus. Dies gilt insbesondere für die gewonnen Selbstständigkeit der Beschäftigten, die zur Entlastung von Fachkräften beiträgt.

## References

[1] Bergold J, Thomas S (2010) Partizipative Forschung. In: Mey G, Mruck K (Hrsg) Handbuch Qualitative Forschung in der Psychologie, 1. Aufl. VS, Wiesbaden, S 333–344

[2] Bethmann A, Hilgenböcker E, Wright M (2019) Partizipative Qualitätsentwicklung in der Prävention und Gesundheitsförderung. In: Tiemann M, Mohokum M (Hrsg) Prävention und Gesundheitsförderung. Springer, Berlin, Heidelberg, S 1–13

[3] Bundesministerium für Arbeit und Soziales (2021) Dritter Teilhabebericht der Bundesregierung über die Lebenslagen von Menschen mit Beeinträchtigungen

[4] Chambers R (2008) PRA, PLA and pluralism: practice and theory. In: Reason P, Bradbury H (Hrsg) The SAGE handbook of action research, 2. Aufl. SAGE, Los Angeles, S 297–318

[5] Clar C, Wright MT (2020) Partizipative Forschung im deutschsprachigen Raum – eine Bestandsaufnahme. Alice Salomon Hochschule Berlin, Berlin

[6] Dias S, Gama A, Simões D et al (2018) Implementation process and impacts of a participatory HIV research project with key populations. Biomed Res Int. 10.1155/2018/5845218 10.1155/2018/5845218 PMC6000914 29955605

[7] Dienel H‑L (2021) Bürgerräte als Konfliktmediatoren – Möglichkeiten, Hindernisse und Beispiele. Die Mediation (Quartal II), S 33–37

[8] Dörnhoff N, Hiller S, Scheiwe N (2012) Zauberwort Partizipation. Lambertus, Freiburg im Breisgau

[9] GKV-Spitzenverband (2020) Leitfaden Prävention

[10] Hartung S, Wihofszky P, Wright MT (2020) Partizipative Forschung – ein Forschungsansatz für Gesundheit und seine Methoden. In: Hartung S, Wihofszky P, Wright MT (Hrsg) Partizipative Forschung. Springer, Wiesbaden, S 1–19

[11] Haveman M, Stöppler R (2014) Gesundheit und Krankheit bei Menschen mit geistiger Behinderung. Kohlhammer, Stuttgart

[12] Hirschberg M (2011) Partizipation – ein Querschnittsanliegen der UN-Behindertenrechtskonvention

[13] Keeley C, Munde V, Schowalter R et al (2019) Partizipativ forschen mit Menschen mit komplexem Unterstützungsbedarf. Teilhabe 58(3):96–102

[14] von Köppen M, Schmidt K, Tiefenthaler S (2020) Mit vulnerablen Gruppen forschen – ein Forschungsprozessmodell als Reflexionshilfe für partizipative Projekte. In: Hartung S, Wihofszky P, Wright MT (Hrsg) Partizipative Forschung. Springer, Wiesbaden, S 21–62

[15] Malterud K, Elvbakken KT (2020) Patients participating as co-researchers in health research: a systematic review of outcomes and experiences. Scand J Public Health 48(6):617–628 31319762 10.1177/1403494819863514

[16] Nieß M (2016) Partizipation. In: Nieß M (Hrsg) Partizipation aus Subjektperspektive. Springer, Wiesbaden, S 67–122

[17] Obermann K, Müller P, Woerns S (2013) Ärztliche Versorgung und Zugang zu ärztlichen Leistungen. Hamburg

[18] PartNet – Netzwerk Partizipative Gesundheitsforschung (2021) Definition – Partizipative Gesundheitsforschung. http://partnet-gesundheit.de/ueber-uns/partnet-definition/. Zugegriffen: 20. Apr. 2021

[19] Straßburger G, Rieger J (Hrsg) (2019) Partizipation kompakt, 2. Aufl. Beltz Juventa, Weinheim

[20] von Unger H (2012) Partizipative Gesundheitsforschung: Wer partizipiert woran? Forum Qual Soc Res 13(1):1

[21] von Unger H (2014) Partizipative Forschung. Springer, Wiesbaden

[22] World Health Organization, World Bank (2011) World report on disability. WHO, Geneva

[23] Wright MT, von Unger H, Block M (2010) Partizipation der Zielgruppe in der Gesundheitsförderung und Prävention. In: Wright MT (Hrsg) Partizipative Qualitätsentwicklung in der Gesundheitsförderung und Prävention, 1. Aufl. Huber, Bern, S 35–52

[24] Wright MT (2021) Partizipative Gesundheitsforschung: Ursprünge und heutiger Stand. Bundesgesundheitsblatt Gesundheitsforschung Gesundheitsschutz 64(2):140–145 33336312 10.1007/s00103-020-03264-y PMC7843534

